# Evidence of Dynamically Dysregulated Gene Expression Pathways in Hyperresponsive B Cells from African American Lupus Patients

**DOI:** 10.1371/journal.pone.0071397

**Published:** 2013-08-15

**Authors:** Igor Dozmorov, Nicolas Dominguez, Andrea L. Sestak, Julie M. Robertson, John B. Harley, Judith A. James, Joel M. Guthridge

**Affiliations:** 1 University of Texas Southwestern Medical Center, Dallas, Texas, United States of America; 2 Oklahoma Medical Research Foundation, Oklahoma City, Oklahoma, United States of America; 3 University of Oklahoma Health Science Center, Oklahoma City, Oklahoma, United States of America; 4 United States Department of Veterans Affairs Medical Center, Cincinnati, Ohio, United States of America; 5 Cincinnati Children’s Hospital Medical Center, Cincinnati, Ohio, United States of America; University of Patras Medical School, Greece

## Abstract

Recent application of gene expression profiling to the immune system has shown a great potential for characterization of complex regulatory processes. It is becoming increasingly important to characterize functional systems through multigene interactions to provide valuable insights into differences between healthy controls and autoimmune patients. Here we apply an original systematic approach to the analysis of changes in regulatory gene interconnections between in Epstein-Barr virus transformed hyperresponsive B cells from SLE patients and normal control B cells. Both traditional analysis of differential gene expression and analysis of the dynamics of gene expression variations were performed in combination to establish model networks of functional gene expression. This Pathway Dysregulation Analysis identified known transcription factors and transcriptional regulators activated uniquely in stimulated B cells from SLE patients.

## Introduction

The use of microarray technology enables one to measure the expressions of tens of thousands of genes simultaneously, allowing an unbiased view of all biological processes with molecular precision. Recent application of gene expression profiling to the immune system has shown a great potential for characterization of complex regulatory processes playing role in any immunological phenomena. Gene expression profiling of inflammatory processes and autoimmune pathologies [1], [2], juvenile rheumatoid arthritis [3], [4], [5], and Sjögren’s syndrome [6] has enhanced our understanding of the delicate balance between a controlled inflammation response and the development of autoimmune disorders.

A more advanced use of microarray analysis focusing on a systems-level analysis represents a powerful method to characterize altered biological systems through dynamic changes of individual gene expression profiles. Advances in microarray technology and systems-level analysis has allowed scientists an opportunity to characterize functional systems through multigene interactions by identifying and characterizing regulatory correlations between individual genes. Identification of functional links between genes responding dynamically to specific treatments through this system biology approach is being increasingly reported in literature [7], [8], [9]. The creative use of gene expression profiling could enable further progress in better understanding the context of abnormalities in signaling pathways in autoimmune patients, especially in systemic lupus erythematosus (SLE).

SLE is a complex disease with heterogeneous clinical features characterized by the production of autoantibodies and the subsequent damage of multiple organ systems. Though the immunological events triggering SLE remain unsolved, a central role for B cells in the SLE pathogenesis has been established in both mice and humans [10], [11], [12]. B cell defects including abnormal function of key signaling molecules, B-cell receptor signaling defects, and perturbations in B cell developmental subsets are hypothesized to play a central role in the breakdown of B cell tolerance and subsequently in SLE pathogenesis.

Research into initiation and pathogenesis of SLE among patients has begun to offer a complex picture of cell signaling and cellular response. A number of cell signaling pathways have been shown to be altered in SLE patients [13], [14], [15], [16]. These include alterations in the interferon pathway [17], TNFα signaling pathway [13], [18], abnormal B cell receptor (BCR) signaling [14], [15], and increased phosphatidylinositol 3-kinase activity [15], [16]. Abnormal cellular responses and cellular populations are also observed in SLE patients. FcγRIIB expression is decreased in the SLE patient memory B cells [19], patient memory B cell subsets are hyper-responsive to stimulation [20], and consist of a large number of transitional B cells [21], [22] and CD19^+^CD24^hi^CD38^hi^ B cells that lack the suppressive regulatory functions observed in controls [23].

In these proof-of-concept experiments, we expand current gene expression profiling methods to apply a systematic approach to the analysis of statistically significant changes in regulatory gene interconnections between in B cells from normal control individuals and the hyperresponsive B cells from SLE patients. We use a novel self-verified experimental design (in which every step, selection, or construction was accepted only when reproduced in duplicated experiments) to identify differentially expressed genes between controls and SLE patients. Our Pathway Dysregulation Analysis identified known transcription factors, genes for inflammatory responses, genes for cell cycle progression, genes for cell growth, genes for response to DNA damage, and genes regulating apoptosis dysregulated in SLE patient derived cell lines.

## Materials and Methods

### Ethics Statement

This study has been conducted according to the principles expressed in the Declaration of Helsinki. These EBV-transformed cell lines were originally generated from systemic lupus erythematosus (SLE) patients and controls as a part of the Lupus Family Registry and Repository and were provided as coded samples for use in this study subject to appropriate IRB approvals at the Oklahoma Medical Research Foundation and the University of Oklahoma Health Sciences Center.

### Cell Culture and in vitro Activation

**Figure 1:**
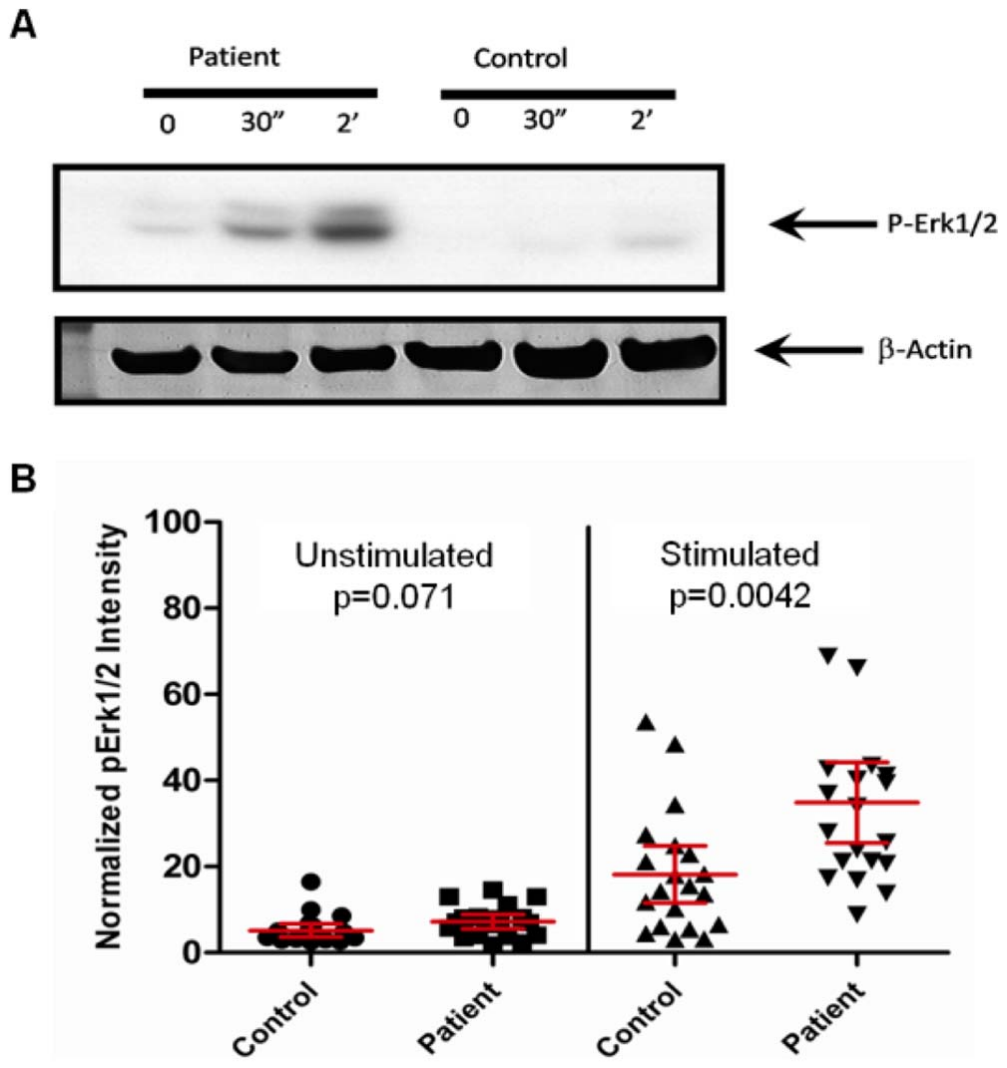
pERK1/2 is upregulated in hyperresponsive B cells after stimulation. B cells isolated from SLE patients and matched controls were stimulated with anti-human IgM F(ab)’_2_ for 30 seconds or 2 minutes. pERK1/2 (A) and normalized pERK1/2 intensity (B) shows increase in hyperresponsive B cells after stimulation.

Potential cell lines from 20 African-American female lupus patients and 20 African-American female controls were screened to identify cell lines that responded to receptor stimulation and exhibited a hyperresponsive B cell phenotype associated with SLE patient B cells. EBV lines were grown under standard culture conditions in RPMI-1640 10% FBS supplemented with l-glutamine, Penicillin-Streptomycin. Cells were washed and cultured at 1×10^6^ cells/ml in serum free media overnight. Goat anti-human-IgM F(ab)’_2_ polyclonal antibody (Jackson ImmunoResearch, Inc.) at 50 µg/ml in RPMI-1640 10% FBS was used to stimulate cells for the indicated periods of time. For signaling experiments, 20 patient and 20 control B cell lines were stimulated for 0.5 or 2 hours and then protein was isolated for Western blotting. The degree of Erk1/2 phosphorylation initiated by anti-IgM F(ab)’_2_ was used to designate each cell line as a “high responder” or a “low responder”, as shown in Figure 1. Although cell lines derived from patients were generally more responsive than those derived from controls, the extremes of the phenotype were used in subsequent experiments, such that the patient lines used were among those most responsive and the control lines used were among those least responsive to BCR stimulation.

For gene expression analysis, a full time course was explored in two high responding patient EBV cell lines and two low responding control EBV cell lines. Time points used in these experiments were 0.5, 1, 2, 4, 8, 16, and 24 hours following stimulation with anti-IgM F(ab)’_2_ antibody. Preliminary analysis of this data revealed that the differences in BCR pathway genes were most evident in the early portion of the time course. Subsequent replication experiments (utilizing another eight cell lines, four SLE lines and four control lines) therefore used time points at baseline, 0.5, and 2 hours only.

### Gene Expression Profiling

Total RNA was isolated from B cells at each time point using RNAqueous total RNA isolation kit (Ambion, Grand Island, NY) and was quantitated by using a Nanodrop scanning spectrophotometer. cRNA was generated and labeled using the Affymetrix cRNA labeling kit (Affymetrix, Santa Clara, CA) according to manufacturer’s specifications. Gene expression was assayed using Affymetrix GeneChip® Human Genome Focus Array representing 8500 verified human sequences from the NCBI RefSeq database according to manufactures specifications.

### Self-verifying Experimental Design

This study’s experimental design is based on duplication of each step of the experimental and analytical procedures. The analysis starts with normalization of expression data followed by selection of hypervariable expressed genes (HVE) distinctive from background at least in one time point. Only genes selected in both of the duplicated experiments are used for further analysis. Verification of the expression profile reproducibility for HVE genes was carried out. Only genes whose expression profiles correlated with the correlation coefficient were used for further analysis. Genes were partitioned into clusters and only the genes gathered into the same clusters in duplicated experiments were used for further analysis. Finally, the networking of HVE genes based on partial correlation is examined and only reproducible edges are presented in the final network.

### Data Analysis

Our methods of data normalization and analysis are based on the use of internal standards that characterize some aspect of system behavior such as technical variability (presented in detail in two recent publications [5], [24]). These methods provide an increase to the power of statistical criterion determined by the content of the internal standard–normally several thousand members–and this enables the use of relatively high statistical thresholds without loss of the sensitivity of the selection. In general, an internal standard is constructed by identifying a large family of similarly behaving genes. Experimental conditions are denoted as an equally expressed cohort. These internal standards were used to robustly estimate parameters that describe some state of the experimental system such as the identification of genes expressed distinctly from background, differentially expressed genes, and genes having similar dynamic behavior. Microarray data was generated in compliance with the Minimal Information About a Microarray Experiment (MIAME) guidelines. This data have been deposited in NCBI’s Gene Expression Omnibus (GEO) database (GEO Submission GSE37573, NCBI tracking system #16567017).

#### Normalization

Normalization for differences among experiments was conducted using a procedure described previously [25]. First, an internal standard is constructed by identifying a set of normally distributed genes having expressions indistinguishable from technological noise [26]. This background cohort enables the statistical selection of genes above or below background with high power. Genes expressed above background are used for unbiased expression levels across arrays by means of a robust regression procedure based on the use of the second internal standard – the equally expressed cohort.

After normalization and adjustment of the gene expression profiles to a common standard, log-transformed gene expressions are used to calculate expression value deviations from the averaged control profile. The log-transformed residuals are mostly independent of expression level and approximate a normal distribution based on the Kolmogorov-Smirnov criterion. Statistical analysis was performed using STATISTICA 6.1(StatSoft, Inc. Tulsa, USA).

#### Internal standard for equity in expression and in variability

The internal standard, or reference group, was constructed by identifying a group of genes which are expressed above background with inherently low variability as determined by an F-test [25], [27]. The reference group presents our third internal standard- technological variation. By creating an estimate of the technological variation, we are able to select a group of biologically stable genes (BSG).

#### Hypervariable expressed genes

Genes whose expression level varied significantly (P<1/N) when comparing an individual gene’s variability to that of the reference group were denoted as HVE [27]. The threshold of P<1/N, N represents the number of genes expressed above background in at least one time point.

#### Associative analysis -identification of differentially expressed genes [25]

These analyses include selections with a Student T-test for replicates using the commonly accepted significance threshold of p<0.05. However, a significant proportion of the genes identified as differentially expressed will be a false positive determination at this threshold level. An associative T-test, in which the replicated residuals for each gene of the experimental group are compared with the entire set of residuals from the reference group defined above, was utilized. The hypotheses are checked if gene expression in experimental group presented as replicated residuals (deviations from averaged control group profile) were associated with very representative (several thousands of members) normally distributed set of residuals of gene expressions in the reference group. The significance threshold was corrected to make the appearance of false positive determinations improbable. Only genes that passed through both tests are presented in the result tables. Genes expressed distinctively from background were determined by analysis of the association of each replicated gene expression with normally distributed background having an average equal to 0 and standard deviation equal to 1. Analysis of the enrichment within gene classifications was assessed using Ingenuity Pathway Analysis (IPA, http://www.ingenuity.com/), Panther Classification System (Panther, http://www.pantherdb.org/), DAVID Bioinformatics Resources 6.7, NIAID/NIH (DAVID, http://david.abcc.ncifcrf.gov/home.jsp) and GeneCards Batch Queries (GeneALaCart, http://www.genecards.org/BatchQueries/index.php).

### Cross-validation of the Selections

A jackknife procedure was used for characterization of the robustness or reproducibility of the differentially expressed genes selection. The comparative analysis was repeated for the two groups of samples with exclusion of one sample from each group throughout the analysis. For two groups with *n* and *m* replicates, *n* × *m* comparisons are possible. Genes selected as differentially expressed in each of these comparisons (selected *n* × *m* times) were ranked as having 100% of reproducibility.

### F-means Cluster Analysis

The clustering procedure consists of the following steps: gene expression normalization, log-transformation and rescaling as noted above; identifying HVE-genes within a group of samples by comparing residual variability of each gene among samples with variability of residuals of all genes in the reference group; and determination of a parameter, termed connectivity, for each of these hyper-variable genes. Connectivity was defined as the number of genes deviated from the seeding profile within ranges determined by the reference group. The p<0.05 threshold for the F-test was used to diminish the proportion of false positive selections.

HVE-genes of each group were sorted by their connectivity and the clustering process was started with genes of highest connectivity. The gene of highest connectivity and all genes whose deviations from expression do not have variability higher than that observed in the reference group are comprised in cluster #1. The next highest gene connectivity and the genes that do not significantly deviate from it comprised cluster #2, and the process continued until all genes were analyzed. Genes that appeared in more than one cluster were considered to be likely functional links among these clusters. Genes with zero connectivity did not belong to any cluster.

### Correlation Mosaic Analysis

The visual presentation of the gene co-expressions based on gene clustering was performed using an original correlation matrix technique as previously described [28]. Comprehensive pair-wise correlations were calculated among gene expression levels such that groups of genes that exhibited temporally correlated behavior could be identified. The clustering procedure was based on the Pearson correlation and follows the same sequence of operations used in the F-means clustering above. However, instead of using variability as a measure of distance, we use the correlation coefficient. The number of clusters and cluster contents are determined by the threshold for correlation coefficient, estimated in simulation experiments. Matrices of correlation coefficients were calculated for these clusters and are represented in a correlation mosaic.

### Gene Networking Based on the Use of Partial Correlation

The method used here was slightly modified from a previously described method [29], [30]. The gene networking analysis was performed in a pair-wise manner for all genes shown to be related in a common cluster. Selection of the environment for each gene (gene **X**) by determining all genes correlated with given one above threshold ***t_e_*** (gene ***X*** environment designated as ***E_x_***). Calculation of a matrix of partial correlations between gene ***X*** and each gene from environment ***E_x_*** with extraction of the influence of all other genes from ***E_x_*** was performed. The ***Y*** row of the matrix consists of partial correlations of given gene ***X*** with some other gene ***YεE_x_*** and with removing the effects of all other genes from ***E_x_*** (each element in the ***Y*** row is partial correlation ***X*** vs ***Y*** (y-row) with withdrawal of the effect of gene ***Z*** from ***E_x_*** (***z***-cell in the ***y***-row)). The partial correlation of variables ***X*** and ***Y*** with respect to ***Z*** was defined as ***pr_xy,z_*** = ***(r_xy_−r_xz_r_yz_)/((1−r_xz_^2^)(1−r_yz_^2^))^1/2^.***

Significance of the partial correlation between any pair of genes (***X*** &***Y*** in above example) is based on the calculation of the minimal ***PC_m_*** among all partial correlations in the row ***y*** and averaged ***PC_a_*** partial correlations between them (for the row ***y***). The partial correlation is recognized as essential if these values exceed thresholds ***t_m_*** and ***t_a_*** correspondingly.

The presence of the only one member (***PC_xy,z_***) in the row below threshold ***t_m_*** was evidence that association between ***X*** and ***Y*** genes is indirect and is a result of their common association with some third gene ***Z***. However the presence analogous “hole” in the row ***z*** (***P_xz,y_<t_m_***) is an evidence for the multiple association between ***X, Y***, and ***Z*** genes (triangle association).

A Monte Carlo simulation study was used to define the statistical thresholds (***t_e_, t_m,_*** and ***t_a_***) below which partial correlation coefficients were likely to be due to chance. For 8-replicated randomized data simulating behavior of 200 genes, using the thresholds ***t_e_*** = 0.8**,** ***t_m_*** = 0.6**_,_** ***and t_a_*** = 0.8 produced less than 5% of false positive selections. The use of duplication increased the sensitivity of the method essentially enabling to decrease these thresholds to the levels ***t_e_*** = 0.6**,** ***t_m_*** = 0.4**_,_** and ***t_a_*** = 0.4 without growth of proportion of the false positive selections.

## Results

### Identification of Hyperactive B cell Lines from Lupus Patients

B cells from lupus patients are known to be hyperresponsive to stimulation through the BCR as evidenced by increased LYN and ERK1/2 phosphorylation [19], [31]. However, it was initially unclear if the same differences would be observed in EBV transformed B cell lines from lupus patients compared to B cell lines derived from unaffected controls. Latent EBV transformation can impact many cell processes, including BCR stimulation [32], [33], [34], [35], but it is unclear whether many of the long-term EBV transformed cells actually express these EBV latent genes. To minimize such influences, we screened potential cell lines from 20 African-American female lupus patients and 20 African-American female controls to identify cell lines which responded to receptor stimulation and exhibited a hyperresponsive B cell phenotype associated with SLE patient B cells.

We assessed the amount of phospho-ERK1/2 generated following BCR stimulation in these EBV transformed cell lines. A representative ERK1/2 phosphorylation response in a case versus a control cell line and normalized phospho-ERK1/2 intensities before stimulation and after stimulation for 2 minutes are shown (Figure 1A and B). Almost all cells responded to stimulation, but the average response was significantly higher in the EBV transformed cells from the lupus cases (based on the results of differential gene expression analysis).

The overarching goal of this research was to assess whether our Pathway Dysregulation Analysis could identify known alterations in the gene expression and signaling pathways of B cells from lupus patients. As such, two control cell lines which were responsive to BCR stimulation and two patient cell lines showing hyperresponsiveness to stimulation were selected for phase I of the study where a complete time-course dynamic expression analysis was performed. Four additional cases and controls were selected in the same way from this screening group to be used in a more limited time-course and focused analysis replication phase of the study where expression at time points 0, 0.5 and 2 hours were analyzed.

### Multi-level Analysis of the Dynamic Response of B cells to Stimulation *in vitro*

#### Experimental data analysis pipeline

**Figure 2:**
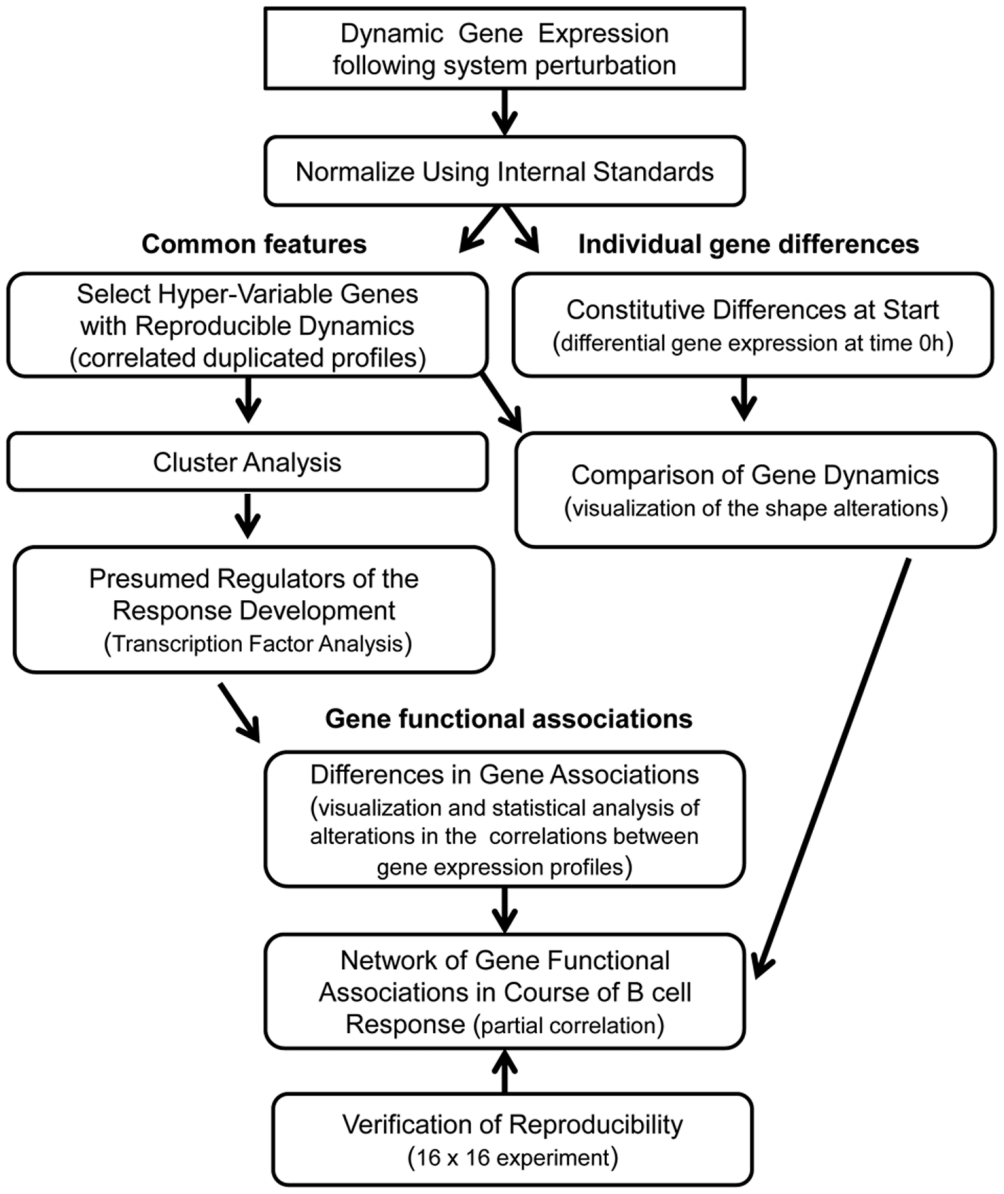
Schematic of pathway dysregulation analysis. The self-verified experimental design of pathway dysregulation analysis is depicted.

We applied a multi-level analysis approach to understand the dynamic response of B cells to stimulation. The first level of analysis identified genes with constitutive differences in expression. These differences were not altered in the course of B cell activation, were able to contribute into activation dynamics as modulating factors creating specific background, and were favorable for differences in dynamic activation in patient B cells. The second level of analysis identified differences in dynamic profiles of expression of individual genes between the two phenotypic groups (gene profiles as independent events). The third level of analysis identified differences in collective behavior of genes associated with changes in gene to gene regulatory interactions. The schematic of this analytical approach is presented in Figure 2. Only reproducible results between the two phenotypic pairs were used in the final analysis. Using this strategy we were able to increase the sensitivity of the statistical tests without the corresponding decrease of specificity and to identify reproducible results in these two independent experimental series within phase I.

### Common Dynamic Expression Responses in Lupus Hyperresponsive B cells Compared to Control B cells

Dynamic gene expression profiles that were independent of the phenotype classifications (patient/control status and B cell hyperresponsiveness) were analyzed. Only genes having statistically significant deviations from the majority of stable genes were selected for the analysis. HVE genes were also assessed between the phenotypic groups. As would be expected, most dynamic gene expression profiles for individual genes were the same following stimulation between the two phenotypic groups. Cluster analysis groups genes based on similar expression profiles and provide clues to the function or regulation of genes. About 500 genes were selected for clustering and networking procedures based on their expression levels (3SD above background at a minimum of one time point) and reproducibility of the expression profiles (replicate phenotype pairs with similar expression profiles and a correlation above 0.8 for each gene in at least one group).

**Figure 3:**
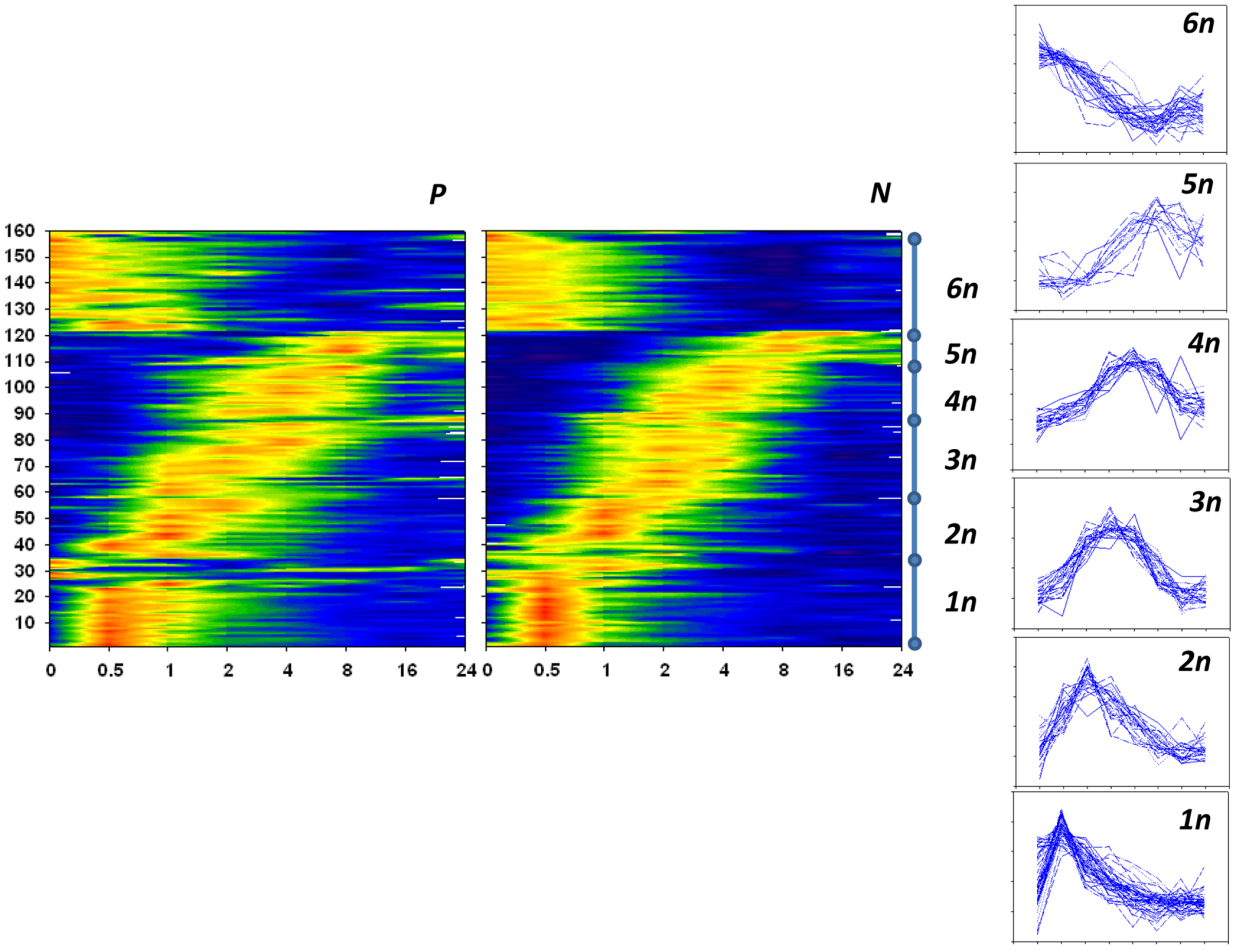
Variable gene clustering after stimulation of B cells from lupus patients and controls. Normalized gene expression data (average = 0, standard deviation = 1) from stimulated hyperresponsive B cells from SLE patients (left) and normal response B cells from control (right). Blue indicates negative normalized expression data and red indicates positive normalized expression data. Six gene clusters and the corresponding cluster profiles are shown to the right side of the heat-maps.

From this analysis, we derived six large clusters containing a total of 160 genes, as shown in Figure 3 (a list of these genes presented in Table S1 in File S1). Clusters are numbered in order of the time of the maximal change in expression of the genes. The six clusters observed included genes which expression was induced during the stimulation protocol followed by a decline in their expression. Cluster #1 presents genes with the earliest (0.5 h) increase of expression and then immediate decline. Cluster #2 shows genes with maximal increase of expression at the 1 h time point with Clusters 3–5 peaking at sequentially later time points and with fewer numbers of genes. Cluster #6 presents genes that had maximal expression before any stimulus was applied and slowly decreased thereafter reaching minimal expression between 16 and 24 hours.

Although the general pattern observed here is the same in all cell lines chosen for study, this general similarity does not exclude significant differences in the behavior of a fraction of the genes between the two phenotypic groups. The genes which do display a difference in dynamic expression profile are described further below.

#### Transcriptional motif analysis within gene expression clusters

**Figure 4:**
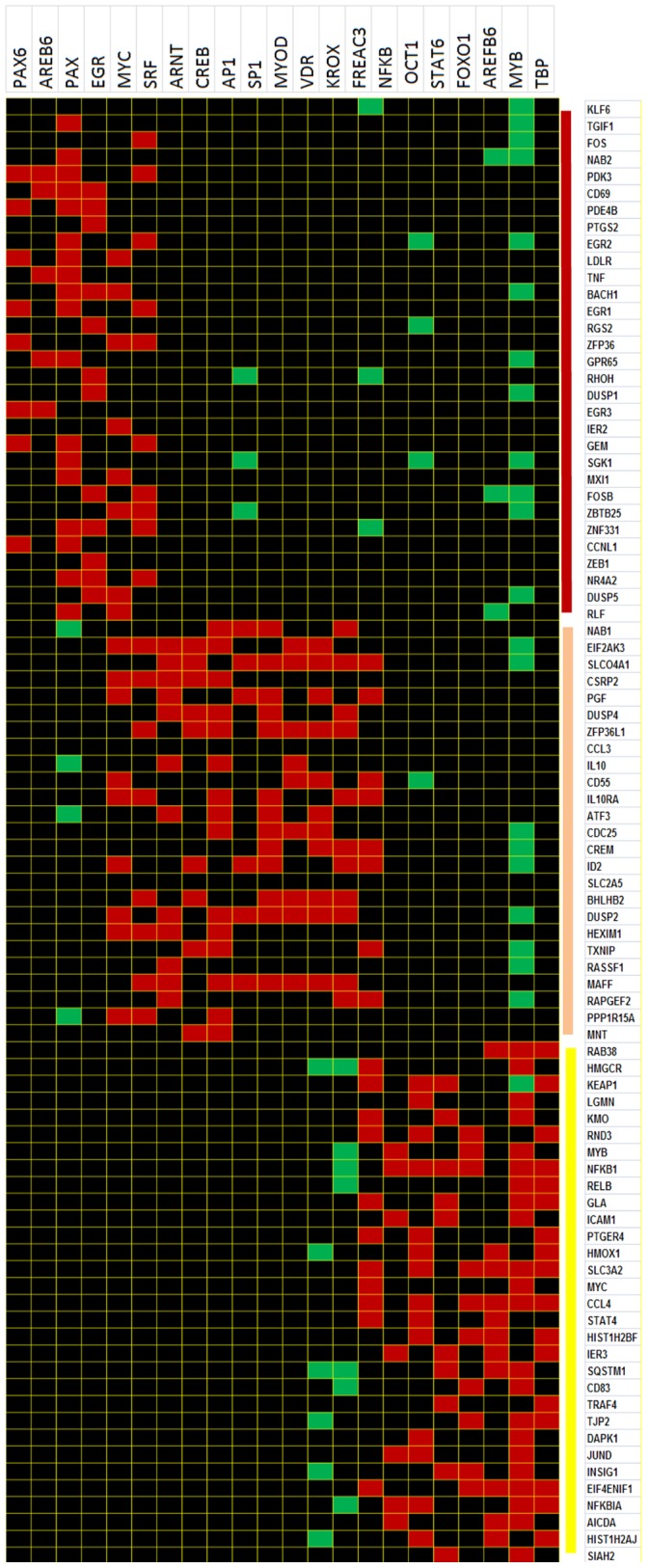
Transcription factor analysis of the variable gene clusters. Transcription factors tested are presented at the top of the diagram. The cluster content is shown along the right. Individual elements of the matrix are colored by the significance of the p-values (threshold p = 0.05): over-representation in the matrix is indicated in red, under-representation is indicated in green.

Analyzing the significance of the individual gene clusters identified by correlated dynamic expression profiles is based upon the assumption that co-expressed genes are likely to share common regulatory motifs [36], [37]. Regulatory motifs common to genes in each of the six clusters were identified using the web-based program PAINT [38]. The predicted list of the transcription factors for the 0.5, 1 and 2 hour time point clusters is shown on the top of Figure 4. A detailed annotation of select transcription factors and their association with known B cell activation events is shown in Table S1 in File S1.

Genes within the clusters and the predicted transcription factors (TFs) responsible for the co-expression of the clustered genes are in agreement with the current B cell activation literature [9], [39], [40], [41] and are detailed in Table S2 in File S1. Analysis of the enrichment within particular gene classifications was next assessed. Cluster 1 mainly consisted of “early response genes” (p = 3.1E^−12^), while clusters 2 and 3 consisted of “regulation of transcription” genes (p = 2.9E^−9^ and p = 1.2E^−6^, respectively).

Many TFs known to regulate B cell genes following activation are identified in this study; however, since these clustered genes represent those which show no differences between the phenotypic groups, the activation signals in the EBV transformed cells cause predictable dynamic expression changes for most B cell genes.

### Individual Gene Differences

While similarities between the expression patterns observed in the EBV-transformed lines exist, specific genes do differ between the phenotypic groups. Utilizing hyperresponsive lupus patient derived cell lines, we sought to identify constitutive differences in gene expression at time 0. Individual gene dynamic expression profiles and/or correlations between multiple gene dynamic profiles that are different between the two phenotypic groups were examined.

#### Constitutive differences in gene expression between phenotypic groups defined by hyperresponsive lupus patient B cells and control B cells

Traditional differential gene expression analysis was carried out at time 0 between SLE patient hyperresponsive B cells and control B cells. This examines the underlying expression signature characteristic to the hyperresponsive state found in patient-derived cell lines. A detailed list, including baseline differences between high-responders and low-responders that regress to the mean over the course of stimulation, is presented in Table S3 in File S2.

Differential gene expression analysis identified initial gene expression differences which are important for regulating cellular responses preceding B cell activation (such as ICAM1, VCAM1, and chemokine receptors). We also observe SLE markers whose association with disease is well known but whose role in B cell activation is not clear such as IRF5, SlamF7 and TLR7.

#### Identification of differences in dynamic gene expression profiles between phenotypic groups

**Figure 5:**
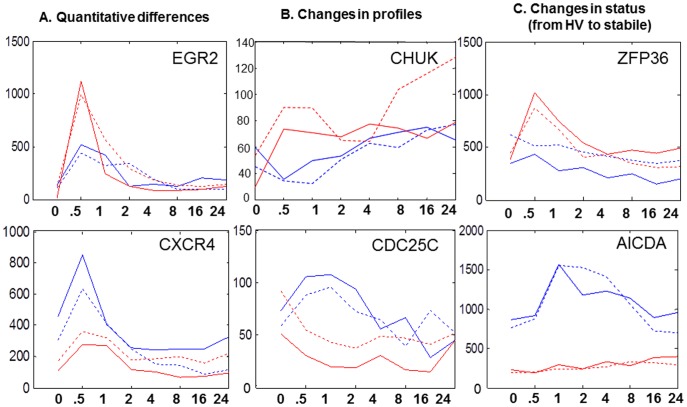
Differences in gene dynamics between normal control SLE samples. The shown profiles are representative of gene dynamics observed in duplicated experiments. Three differences in gene dynamics are shown: quantitative differences (left), changes in gene profiles (middle), and changes from hyper variable to stable (right). Graphs were shown as hours after stimulation (x-axis) and normalized gene expressions (y-axis). Each line on the graph represents one cell line. Each cell line was classified as a high responder (solid line) or low responder (hatched line). SLE patient sample gene expression is shown in red; while normal control sample gene expression is shown in blue.

A simple clustering analysis reveals, as expected, a very high level of similarity in the dynamic expression profile of most genes. However, discreet differences in the dynamic expression profiles for select genes were found. We were able to discriminate between three types of gene expression dynamics between the high and low B cell activation response groups as outlined in Figure 5. These differences in gene expression dynamics can reflect quantitative differences in the level of expression despite similar profile patterns, changes in association between genes, and gene clusters. Examples of these differences are given in Figure 5 and in Table S3 in File S2. Interestingly, these include genes previously associated with B cell activation and SLE. Since our phenotypic groups are based upon differences in B cell responsiveness to stimulation, it is encouraging that we were able to identify these enhanced responses in dynamic profiles of early response genes.

**Figure 6:**
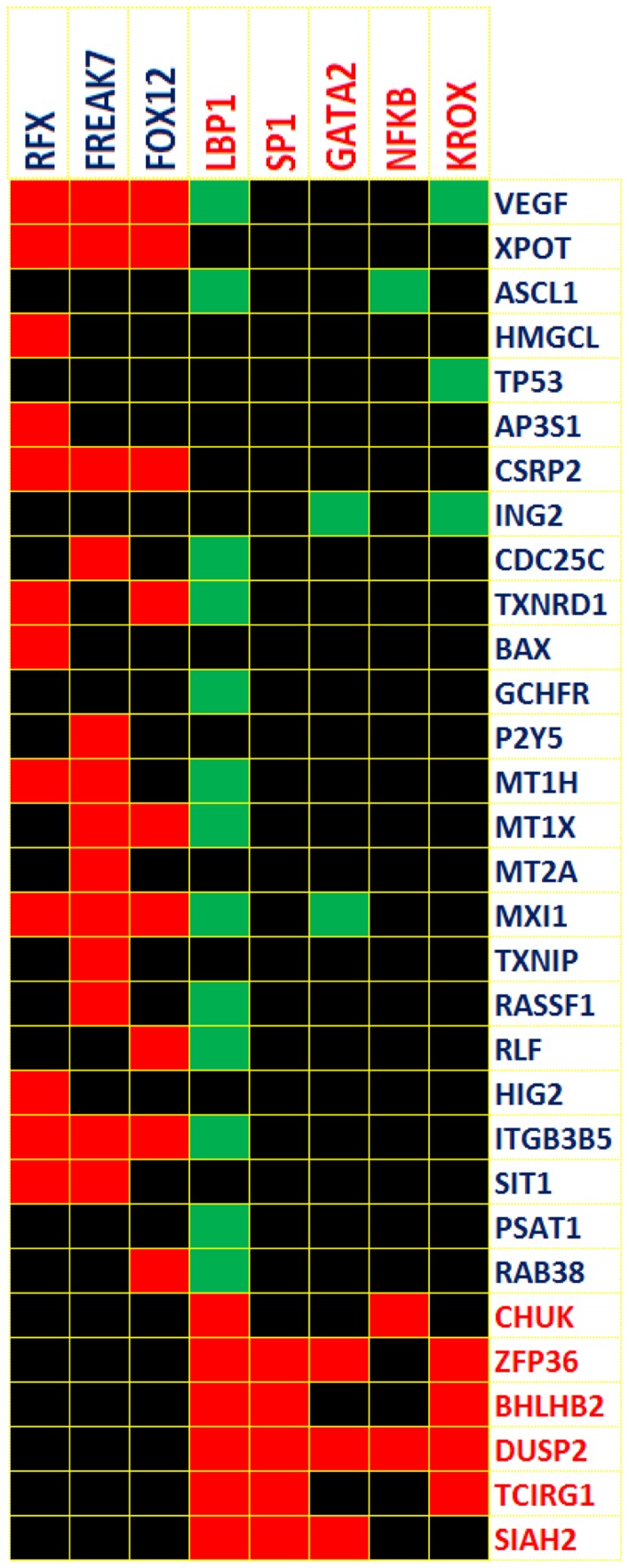
Transcription factor analysis of uniquely activated genes in control and SLE patient samples. The transcription factors tested are shown at the top of the figure. Blue text represents unique genes identified in control samples; while red text identifies unique genes identified in SLE patient samples. Individual elements of the matrix are colored by the significance of the p-values (threshold p = 0.05): over-representation in the matrix is indicated in red, under-representation is indicated in green.

Several genes demonstrated very unique profiles of dynamic expression responses following B cell stimulation. While these genes have not previously been associated with SLE, they might warrant further investigation as potential candidate genes. These include genes such as ZBTB25, AICDA, HMGCL, PGF, HIG2, HRSP12, GCHFR, and LGMN. Each of these genes was only activated in normal responsive B cells. In contrast, some genes such as GPR137B, NAB1, and TCIRG1 were only expressed in hyperresponsive B cells from SLE patients. Transcription motif analysis for such genes uniquely variable in only one subgroup is shown in Figure 6.

### Evidence of Potential Functional Associations between Genes Based Upon Dynamic Gene Expression Data

#### Analysis of differences in gene functional associations

**Figure 7:**
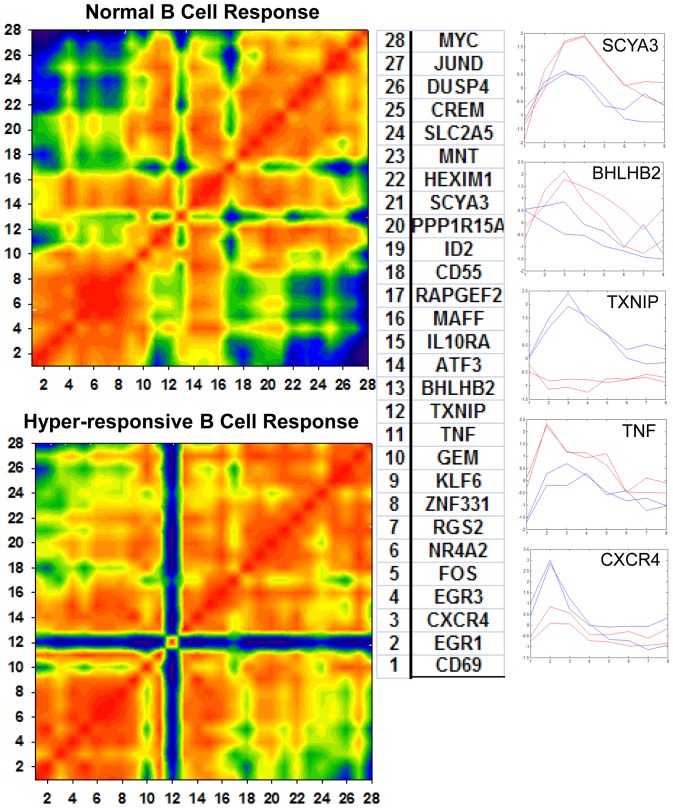
Correlative gene associations in normal B cell responses and in hyperresponsive B cell responses. Pearson correlation was utilized to estimate the correlation coefficients. Negative correlations are shown in blue; while positive correlations are shown in red. Genes examined are listed in table on the left. Gene numbers (right column) are used as coordinates along x and y-axis. Select gene expression graphs are shown on the far right with the two SLE patient cell lines depicted in red and the two control cell lines depicted in blue. The order of genes is maintained giving clear visualization of differences in gene associations.

In the results presented above, we have outlined three types of dynamics of gene expression. Of these, changes in dynamic expression profiles and expression of genes exclusively in one phenotype group can clearly be visualized using a correlative mosaic presentation [28] as shown in Figure 7.

Correlative mosaics yield information about all gene-to-gene associations within the functionally defined group of genes and enables direct visual comparison of these associations between the two phenotypes. Genes with similar profiles tended to cluster together. Correlations were very high between genes within the same cluster, suggesting a functional link [42].

Comparison of the correlation mosaic between the B cell control samples and the hyperresponsive B cells derived from lupus patients identifies regulatory associations that are different between the two groups. In hyperresponsive B cells, significantly changed dynamic expression profiles are easily observed. Other patterns of dynamic expression, including genes whose pattern of expression dynamics is similar between the phenotypic groups but whose quantitative levels of expression differ, can also be easily observed. These genes can not necessarily be excluded from having a functional association difference because of the lack of correlation differences in this type of visualization.

#### Use of discrete time periods of a dynamic gene expression response to reconstruct networks of gene functional associations

**Figure 8:**
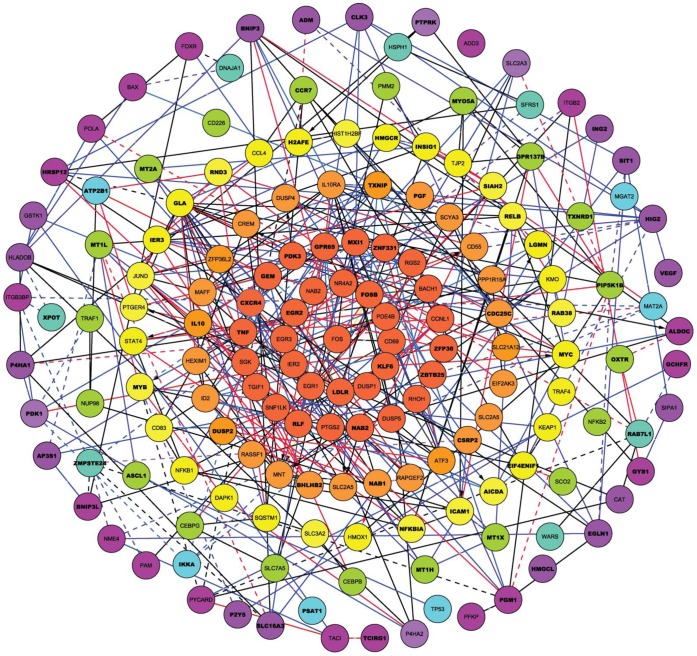
Gene network interaction after B cell stimulation of SLE patient and normal control samples. A gene network interaction map (from the initial analysis group) built using the partial correlations method is shown. Genes were grouped in different colored clusters representing maximum expression levels at various time points after B cell stimulation. The center gene clusters represent genes with maximum expression levels obtained after 0.5 hours of stimulation; then followed by colored circles for genes with maximum expression levels at 1, 2, 4, 8 hours, while the genes in peripheral circle reach maximum expression levels 16 to 24 hours after stimulation. Blue lines linking genes represent gene associations found in normal control samples. Red lines represent gene associations found in SLE patient samples. Black lines indicate gene associations found in both groups. Dashed lines represent negative gene associations.

The correlative clustering presented in Figure 7 partitions genes with similar expression dynamics into highly correlated groups. However, not every pair of highly correlated genes is necessarily functionally interconnected. As such, we next utilized partial correlation coefficients which are better able to identify direct functional interconnections and tend to exclude third party influences [43]. The unique gene-gene regulatory associations that appear in only one phenotypic group were obtained from a paired analysis. Partial correlations were calculated for each pair of genes. If significant partial correlations appeared for any given pair of genes simultaneously in both independent experiments, this was considered evidence for the functional association between these genes. The resulting associations are visualized by the network shown in Figure 8. This figure reveals groups of genes which likely share a common biological process at the peak time interval where the cluster’s genes are expressed. Networking gives additional information about cause-effect or temporal nature of a relationship between genes. Detailed information about all gene interconnections is given in Table S1 in File S1.

#### Phenotype specific sub-networks focused on B cell activation, apoptosis or lupus associated genes

**Figure 9:**
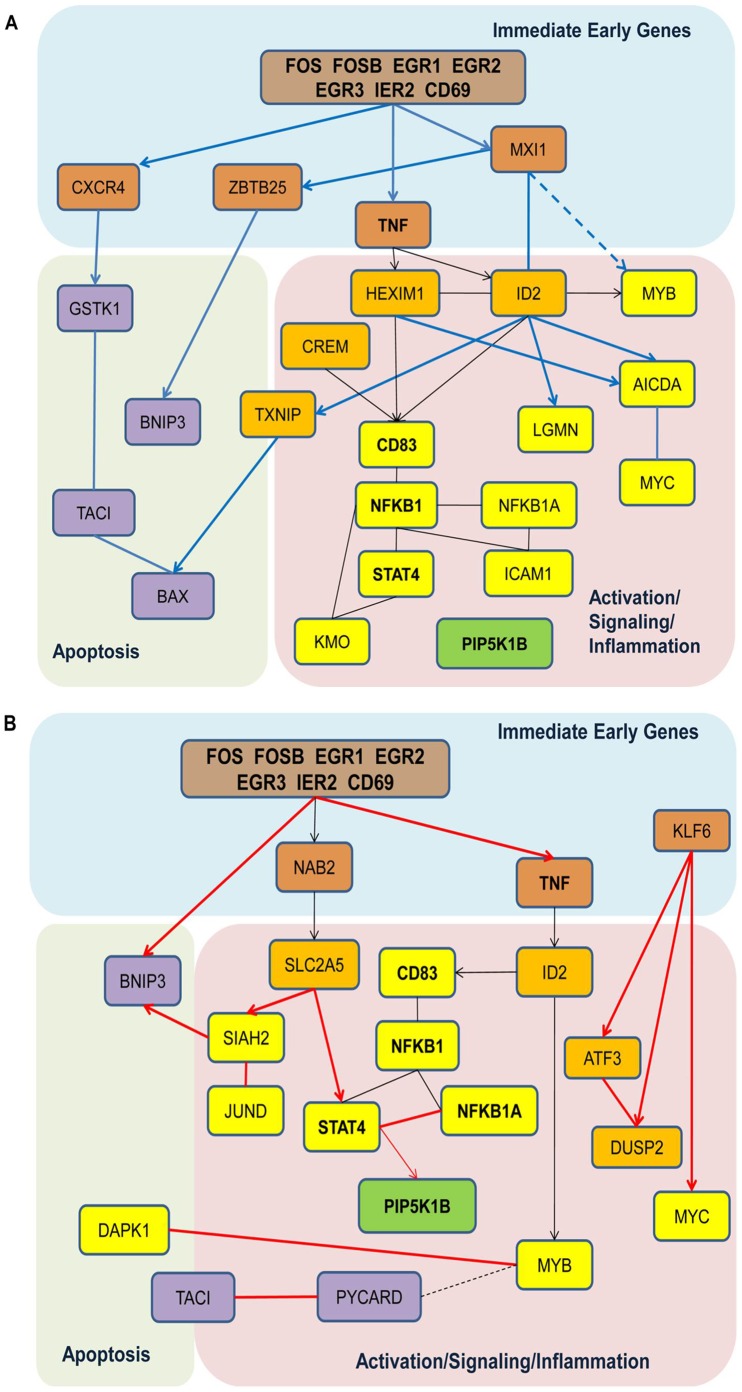
Dynamic gene networks in normal and SLE patient B cells. Unique dynamic gene networks of the examined (A) normal (n = 2) and (B) hyperresponsive B (n = 2) cells are shown. Genes that appear in both the normal and hyperresponsive B cells are in bold.

If we focus on genes that demonstrate statistically significant dynamic behavior in one phenotypic group while remaining stable or not expressed in another phenotypic group we can gain insight into potentially functional genes and their interconnections in pathways. Figure 9A presents fragments of the dynamic expression network we constructed in Figure 8, where a majority of the genes display a prominent dynamic behavior only in the normally responsive phenotypic group derived from controls. Based upon information presented in Table S3 in File S2, all of these genes fall into two functional categories: regulatory control of the B cell activation and pro-apoptotic activity. In this analysis, these very early response genes were correlated with the expression of inflammatory, cytokine and chemokine receptor genes in samples from normally responsive control B cells. Connections to genes downstream of B cell activation and inflammation were observed. In addition, interconnections between the early activation genes and downstream apoptosis related pathway genes exist and were only observed in the normal responsive B cells from control subjects (Figure 9A).

In contrast, several interconnections are found between genes based on correlated expression which are only observed in hyperresponsive B cells (Figure 9B). While several of the gene nodes in the activation/signaling/inflammation pathway are similar between the normal responsive and hyperresponsive B cells, it is the correlations between these genes that differ. Only a few genes in the apoptosis pathway appear to be correlated with the upstream early activation genes, suggesting that the apoptosis pathway regulation is different between the two phenotypic groups.

#### Confirmation analysis

While the results presented thus far in two independent dynamic expression experiments unambiguously demonstrate reproducibility, we carried out an additional experimental series with 8 additional pairs of cell lines characterized as normal responsive and hyperresponsive. We only replicated the most informative time points (0 and 0.5 hours) and were able to demonstrate confirmation of approximately 80% of earlier established differences (See Table S2 in File S1). The analysis of dynamic changes in gene expressions between the two earliest time points (0 and 0.5 hr) confirmed the same increase of expression of most genes from cluster 1 in Figure 4 with the exception of EGR4 (data not shown).

## Discussion

Gene expression profiling by microarray analysis has revolutionized the study of biology by allowing for the simultaneous examination of thousands of genes. However, the ultimate goal is to characterize details of the studied system by identifying functional associations between its genes.

Here we use a systems biology approach in proof-of-concept experiments to analyze differences in gene functional associations in B lymphocytes. B cells from two different phenotypically discrete groups, normally responsive B cells derived from African-American healthy donors and hyperresponsive B cells derived African-American lupus patients were used to identify differences in the regulation of sub-networks characteristic for the B cell response. We employed a multistage analysis collectively called the Pathway Dysregulation Analysis. The first step established genes that responded to B cell activation irrespective of the phenotypic group. The sequences of changes in expression, or the dynamic profiles, were clustered to identify groups of genes with similar functionality. The next step in the analysis was to establish constitutive differences in gene expression between the phenotypic groups. The third step was to identify genes where differences in the dynamic expression profile could discriminate between the phenotypic groups. The final step was to create a model reflecting the complexity of the functional associations between genes in the network and allow one to visualize unique features of the interconnections.

A critical caveat of this approach, discriminating it from many similar analyses, is that the course is determined in part by the reproducibility of the data. First, all of the data (phenotype group 1 (patient), group 2 (control), stimulated and unstimulated) were normalized together, thereby minimizing the systemic differences within and between groups of samples (Level 1). Next, genes were selected on the basis of their variability in the system; those with significantly extended variation compared with the majority of genes (which were stable both between both phenotypic groups and under stimulatory conditions) (Level 2). This step demanded replication; only HVE-genes with highly correlated profiles in duplicated subgroups (cc>0.8) were used for subsequent analysis.

We cannot conclude that the differences seen between the two phenotypic groups are necessarily representative of all SLE patients and all controls as our study was focused on assessing whether the Pathway Dysregulation Analysis method would identify genes/pathways indicative of the reported biology. As such, our experiments were not powered to fully test dynamic gene expression differences between lupus patient and healthy control B cells. However, differences in the dynamics of gene expression following B cell activation between these two phenotypic groups can give some insights into the genes and pathways which are potentially different between these two groups.

Other multivariate methods of analysis show potential to rapidly identify genes and groups of genes involved in normal and disease processes. However, there are practically no examples of reproduction of the sophisticated constructions created for the explanation of biological phenomena. A well-recognized problem with multivariate analyses in microarray experiments is reproducibility and verification [44]. To reduce this concern, we utilized an experimental design with reproducibility testing at each level of analysis.

The final results of our analysis include a very complicated network of functional associations of many genes, but the details of this complex network can be replicated by two independent experiments. While this is of course encouraging, it is still difficult to ascertain functional consequences of discrete differences in such complicated networks. Our approach takes advantage of incorporating additional parameters derived from analysis of the differences in the individual gene dynamics, contents of the clusters, gene functional interconnections in the network, and comparison of this information with prior knowledge about B cell activation and gene expression dynamics. Numerous approaches can create gene networks through reverse engineering methods. However, most of these methods rely upon correlation or other measures of association and are unable to discriminate between direct and indirect associations [43]. Our technique is based on the use of partial correlation which is able to discriminate between direct and indirect associations similar to the method applied in series of publications on the ARACNe method (Data Processing Inequality method in [9], [45], [46], [47]). More informative associations can be obtained with methods based on the estimation of the probabilities of associations and mutual information ([9], [45], [48], [49], [50], however, these methods need much more information (more replicates or time points) to establish reliable associations. These methods are more sensitive to experimental errors and depend upon categorizing the data into a discrete binary parameter (e.g. expressed/not expressed states) for entropy calculation.

It is important to be able to discriminate between the biologically driven variations of gene expression and the chaotic noise type variations that are very common for microarray experiments. The approaches usually employed are based on subjectively established thresholds for this discrimination [9]. In our analysis we employed the selection of hyper-variably expressed genes. This is based upon the use of the internal standard methodology and application of strong statistical criteria to enable high power and high specificity selections of genes. Application of an additional filter to the selection of genes in the dynamic analysis is based upon the use of reproducibility of their dynamic expression profiles. Variations due to background noise are usually not reproducible.

Our study demonstrates that multilevel analysis is capable of defining gene regulatory pathways which not only reflect the differences in hyper vs. normal B cell responsiveness to activation, but also represents candidate pathways that may be the target of functional dysregulation in diseases with hyperresponsive B cells, such as lupus.

## Supporting Information

File S1 Table S1, Cluster allocations and functional interconnection of 160 hyper-variably expressed genes in response to B cell stimulation. Data is a composite of the initial analysis group consisting of two control and two SLE patient cell lines. Table S2, Differentially expressed genes between normal and SLE-patient samples at early time points after B cell stimulation. Columns A and B describe information about the genes in the array. Columns C–H and I–M show normalized levels of gene expression (mean and standard deviation) for normal control and SLE-patient samples. Columns G and M depict the p-value obtained from an associative test, while columns H and N have the ratio of patient to control expression levels. The results of analysis are shown in columns O–R. In columns O–R, the minimum expression level set at >20 is used as the requirement for gene expression to be considered above background. In columns O and P, minimum differences in fold expression changes is set at >2. In columns Q and R the restriction for fold differences is decreased to 1.3. Expression Changes designations: A1 = significant overexpression in patient samples versus control samples; A2 = significant overexpression in control samples versus patient samples; A3 = gene expressed only in patient samples; A4 = gene expressed in only control samples. Columns S-AF show the results of the confirmatory analysis using the same samples as the initial analysis. Data from the initial two SLE patient and two 2 control cell lines are shown.
(XLSX)

File S2 Table S3, Differences in dynamic gene expression profiles between phenotypic groups. Genes marked with blue are overexpressed in normal control samples, marked with red are overexpressed in pathological samples, **bold** symbols are used for genes uniquely dynamical in one group and stabile or not expressed in another. The lines on the graph are representative of the two SLE patient (red) and two control (blue) cell lines initially examined. Each phenotypic group was split into a high responder (solid) or low responder (hatched) cell line based on response to BCR stimulation.
(DOC)
