# Supplementary material for: Evidence of Dynamically Dysregulated Gene Expression Pathways in Hyperresponsive B Cells from African American Lupus Patients
Source: PLoS One. 2013 Aug 15;8(8):e71397. doi: 10.1371/journal.pone.0071397 (PMC3744560; doi:10.1371/journal.pone.0071397)
Supplement: File S2 — Table S3, Differences in dynamic gene expression profiles between phenotypic groups. Genes marked with blue are overexpressed in normal control samples, marked with red are overexpressed in pathological samples, bold symbols are used for genes uniquely dynamical in one group and stabile or not expressed in another. The lines on the graph are representative of the two SLE patient (red) and two control (blue) cell lines initially examined. Each phenotypic group was split into a high responder (solid) or low responder (hatched) cell line based on response to BCR stimulation. (DOC) [file pone.0071397.s002.doc]

**Evidence of Dynamically Dysregulated Gene Expression Pathways in**

**Hyperresponsive B Cells from African American Lupus Patients**

**Supplementary Material**

Igor Dozmorov^1^, Nicolas Dominguez^2^, Andrea L. Sestak^3^, Julie M. Robertson^1^,

John B Harley^4,5^, Judith A James^2,3^, Joel M Guthridge^2*^

^1^UT Southwestern Medical Center, Dallas, TX, 75390, USA, ^2^Oklahoma Medical Research Foundation, Oklahoma City, OK, 73104, USA ^3^ University of Oklahoma Health Science Center, Oklahoma City, OK, 73104, USA ^4^US Department of Veterans Affairs Medical Center, Cincinnati, OH, 45220, USA ^5^Cincinnati Children’s Hospital Medical Center, Cincinnati, OH, 45229, USA.

Key words: systemic lupus erythematosus, microarray, B cell pathways

Corresponding author:

Joel Guthridge, PhD

Oklahoma Medical Research Foundation

825 NE 13th Street, MS#53,

Oklahoma City, OK 73104

E-mail: Joel-Guthridge@omrf.org

Telephone: 405 271-2600

Fax: 405 271-7063

**Supplementary Table 1.** Cluster allocations and functional interconnection of 160 hyper-variably expressed genes in response to B cell stimulation. Data is a composite of the initial analysis group consisting of two control and two SLE patient cell lines.

See File S1

**Supplementary Table 2.** Differentially expressed genes between normal and SLE-patient samples at early time points after B cell stimulation.

Columns A and B describe information about the genes in the array. Columns C-H and I-M show normalized levels of gene expression (mean and standard deviation) for normal control and SLE-patient samples. Columns G and M depict the p-value obtained from an associative test, while columns H and N have the ratio of patient to control expression levels. The results of analysis are shown in columns O-R. In columns O-R, the minimum expression level set at >20 is used as the requirement for gene expression to be considered above background. In columns O and P, minimum differences in fold expression changes is set at >2. In columns Q and R the restriction for fold differences is decreased to 1.3. Expression Changes designations: A1= significant overexpression in patient samples versus control samples; A2= significant overexpression in control samples versus patient samples; A3= gene expressed only in patient samples; A4= gene expressed in only control samples. Columns S-AF show the results of the confirmatory analysis using the same samples as the initial analysis. Data from the initial two SLE patient and two 2 control cell lines are shown.

See File S1.

**Supplementary Table 3.** Differences in dynamic gene expression profiles between phenotypic groups. Genes marked with blue are overexpressed in normal control samples, marked with red are overexpressed in pathological samples, **bold** symbols are used for genes uniquely dynamic in one group and stabile or not expressed in another. The lines on the graph are representative of the two SLE patient (red) and two control (blue) cell lines initially examined. Each phenotypic group was split into a high responder (solid) or low responder (hatched) cell line based on response to BCR stimulation.

| 1. **Genes associated with lupus involved in B cell activation** | | | |
| --- | --- | --- | --- |
| - ***Genes upregulated in lupus samples***   According to well-established B cell hyperactivity in systemic lupus erythematosus, we can expect enhanced responsiveness to BCR stimulation of B cells from lupus patients compared to normal controls (64). We can see these enhanced responses in dynamic gene expression profiles of early response genes (EGR2, FOSB, TNF, KLF6, CCR7 etc). | | | |
| EGR2 | | Pro-mitotic and anti-apoptotic gene of the early growth response family  B cell early response gene. Blockade of EGR2 activity elucidated a dose-dependent inhibition of B cell activation/proliferation. This gene may represent a point of convergence in the integration of different signaling pathways into the B cell proliferative response . |  |
| FOSB | | Stimulation of B cells leads to a rapid and transient increase in fosB mRNA levels. FosB plays a central role in regulating gene expression during B cell activation. . |  |
| TNF | | This cytokine is involved in the regulation of a wide spectrum of biological processes including cell proliferation, differentiation, apoptosis, lipid metabolism, and coagulation. This cytokine has been implicated in a variety of diseases, including autoimmune diseases |  |
| CCR7 | | The chemokine receptor CCR7 is a key organizer of the immune system. CCR7-dependent interplay of the three cell-types (T,B,DC) is crucial for virus-neutralizing B cell responses in the presence of limiting amounts of Ag . |  |
| **ZFP36**  The immediate early protein tristetraprolin (TTP) | | In parallel with TNF dynamics, there is activation of the **ZFP36** gene (only in lupus patient cells) that can bind and destabilize the TNF-alpha mRNA . This gene is also responsible for the intrinsic B lymphocyte defects in Ig class switch recombination (it was shown only for aged B lymphocytes . ZFP36 might influence TNF-alpha regulation at multiple levels. |  |
| NFKBIA | | Nuclear factor kappa B (NFkappaB) is an important transcription factor that together with its inhibitor (IkappaB) participates in the activation of genes involved in immune responses. Additionally, 152 patients with systemic lupus erythematosus (SLE) were genotyped for NFKBIA polymorphism . |  |
| **DUSP2** | | DUSP2 (encoding the phosphatase PAC-1) is one of the most highly induced transcripts in activated immune cells. Genetic cancellation of DUSP2 expression in Dusp2 (-/-) mice considerably reduced inflammatory responses in the model of rheumatoid arthritis . This gene serves to negatively regulate members of the mitogen-activated protein (MAP) kinase superfamily (MAPK/ERK, SAPK/JNK, p38), which are associated with cellular proliferation and differentiation. |  |
| **GSTK1**  Gluta-thione S-trans-ferase kappa 1 | | Involved in the Aryl Hydrocarbon Receptor Signaling. There was some observed association between SLE diagnosis and glutation S-transferase gene variants . |  |
| **IL10** | | Regulatory B cells, including those that produce interleukin-10 (B10 cells), function as negative regulators of inflammatory immune responses . |  |
| - ***Genes not expressed or stabile in patient samples, but dynamically active in healthy control samples*** | | | |
| **TACI** | **TNFRSF13B – TACI** - is a lymphocyte-specific member of the tumor necrosis factor (TNF) receptor superfamily. Regulated by TLR9, MYD88 and others. **Loss of TACI causes fatal lymphoproliferation and autoimmunity**, establishing TACI as an inhibitory BLyS receptor. Treatment of B cells with TACI agonistic antibodies inhibits proliferation in vitro and activation of a chimeric receptor containing the TACI intracellular domain induces apoptosis. These results demonstrate the critical requirement for TACI in regulating B cell homeostasis.  TACI activation in a B cell line results in nuclear factor kappaB and c-Jun NH(2)-terminal kinase activation. The identification and characterization of the receptor for TALL-1 provides useful information for the development of a treatment for B cell-mediated autoimmune diseases such as systemic lupus erythematosus . | |  |
| CXCR4 | CXCR4 possesses a unique response profile and distinguishes itself through a prolonged signaling capacity .  B cell activation upregulates CXCR4.  Expression of CXCR4 was significantly reduced in all SLE subsets compared with normal.  B cell activation upregulates CXCR4 . In our case the transitory upregulation is seen in both cases though at higher degree in normal control. | |  |
| ALDOC | Statistically significant decrease of ALDOC activity was found in the groups of patients with chronic proliferative glomerulonephritis, membranoproliferative glomerulonephritis, membranous glomerulonephritis and lupus nephritis in comparison with the healthy individuals from the control group . | |  |
| **ASCL1**  (MASH1) | Notch signaling pathway. Notch activity synergizes with B-cell-receptor and CD40 signaling to enhance B-cell activation | |  |
| 1. **Differences in healthy controls**   B cell activation enables to reveal detailed control of many dynamical processes involved in activation and to see essential differences associated with pathology. Many genes playing critical control role appeared to be active in only normal cells and remain non-responsive or even not expressed in lupus B cells. Within them there are genes controlling cell cycle (CDC25C; PDK1,3; ), receptor signaling (TEC), hypermutations and Ig switch (AICDA). | | | |
| - ***Controls that are expressed in healthy controls only*** | | | |
| **CDC25C** | This gene plays a critical role in the control of the cell cycle and in the checkpoint response to DNA damage. It directs dephosphorylation of cyclin B-bound CDC2 and triggers entry into mitosis . The Cdc25A is required both for progression through mitosis and for Myc-induced apoptosis, making cdc25A the most attractive Myc target gene identified so far . | |  |
| **AICDA** | Activation-induced deaminase initiates somatic hypermutation - introduces point mutations into immunoglobulin genes in germinal center B cells during an immune response. The observed over-expression of the AICDA can be associated with EBV infection of B cells . | |  |
|  | | | |
| **MXI1** | MYC antagonist. As a members of the Myc oncoprotein network (c-Myc, Max, and Mad) this gene plays important role in proliferation, differentiation, and apoptosis . | |  |
| **RLF**  L-myc fusion sequence (RLF) | L-Myc is a potent activator of several basally expressed MYC family oncoproteins . | |  |
| B-MYB | The protein is a nuclear protein involved in cell cycle progression and a member of the MYB family of transcription factor genes,. The encoded protein is phosphorylated by cyclin A/cyclin-dependent kinase 2 during the S-phase of the cell cycle and possesses both activator and repressor activities. It has been shown to activate the cell division cycle 2, cyclin D1, and insulin-like growth factor-binding protein 5 genes. Transcript variants may exist for this gene. | |  |
| GEM | The protein encoded by this gene belongs to the RAD/GEM family of GTP-binding proteins. It is associated with the inner face of the plasma membrane and could play a role as a regulatory protein in receptor-mediated signal transduction . It plays an important role in the cell functions, including contractility, chemotaxis, adhesion, and migration . | |  |
| PDK1 | PDK1 plays an important role in cell proliferation and cell cycle progression through control of cyclin D1 and p27Kip1 expression . | |  |
| **CSRP2** | CSRP2 is a member of the CSRP family of genes, encoding a group of LIM domain proteins, which may be involved in regulatory processes important for development and cellular differentiation. | |  |
|  | | | |
| **MT1L** | Metallothionein (MT) is a protein inducible by heavy metals, various chemicals, immunomodulatory substances, and interleukins. MT exerts a proapoptotic function . Individuals that develop an inflammatory autoimmune disease in the a**b**sence of adequate MT levels may experience a more severe form of the disease than they might in the presence of MT. There are several reports that serum MT levels are altered in human patients with certain forms of autoimmune disease. For example, serum MT levels are decreased below normal in individuals with rheumatoid arthritis and systemic lupus erythematosus . | |  |
| **MT1H** | metallothionein 1 H (MT1H), a hypoxic and oxidative stress protein.  MMPs, proinflammatory cytokines, and oxidative stress-derived molecules  See MT1X | |  |
| **MT1X** | Stress response proteins can play integral roles as modulators of cellular function and can be involved in mechanisms that are important to immune function. Metallothioneins (MT), a cysteine-rich stress response protein, has been shown to play numerous roles in the cell and participate in modulating immune responses by regulation of transcription factor activity . | |  |
| **MT2A** | Metallothioneins are proteins inducible by heavy metals, various chemicals, immunomodulatory substances, and interleukins. MT exert a proapoptotic function . | |  |
|  | | | |
| **SIAH2** | TRAF2, serves as a central regulator of the cellular response to stress and cytokines through the regulation of key stress-signaling cascades. SIAH2 decreases stability of TRAF2 under stress conditions . Upregulation of the SIAH2 gene was observed in pathological cells only. | |  |
| 1. **Apoptosis associated differences**   Most genes demonstrating significant differences in dynamical behavior after B cell stimulation are involved in apoptosis regulation. Most genes carrying out pro-apoptotic functions are dynamically active only in normal samples whereas genes with anti-apoptotic function are active in pathological samples only. | | | |
| - ***Genes upregulated in healthy control samples*:**   Above presented metallothioneins (**MT1L**, **MT1H**, **MT1X, MT2A**) exert a proapoptotic function (86). | | | |
| GPR65  TDAG8 | This gene encodes a pro-apoptotic G protein-coupled receptor whose activation promotes glucocorticoid-induced apoptosis . | |  |
| **MXI1** | MYC antagonist. As a members of the Myc oncoprotein network (c-Myc, Max, and Mad) this gene plays important role in proliferation, differentiation, and apoptosis. Pro-apoptotic . | |  |
| **RAB38** | RAB38 plays a role in the sorting of TYRP1, tyrosinase related protein with established potential role in apoptosis regulation . | |  |
| MYB | c-Maf can interact with c-Myb to reduce Bcl-2 expression, thereby limiting cell survival following receptor engagement. | |  |
| **TXNIP** | TXNIP is required to maintain sufficient thioredoxin NADPH activity to reductively reactivate oxidized PTEN and oppose Akt downstream signaling. TXNIP upregulation results in ASK1 activation, ER stress, and p38 and JNK phosphorylation, all leading to apoptosis.  TXNIP deficiency induces Akt/Bcl-xL signaling and inhibits apoptosis of pancreatic beta-cells . | |  |
| **TXNRD1** | Downregulation leads to a significant decrease of apoptosis . | |  |
| ING2 | This gene is a member of the inhibitor of growth (ING) family. Members of the ING family associate with and modulate the activity of histone acetyltransferase (HAT) and histone deacetylase (HDAC) complexes and function in DNA repair and apoptosis. Some ING proteins are involved in transcriptional regulation of genes, such as the p53-inducible genes p21 and Bax. ING proteins are able to restrict cell growth and proliferation, induce apoptosis, and modulate cell cycle progression . | |  |
| BNIP3 | Actively involved in promoting apoptosis by inducing the expression of death receptor ligands, including Fas ligand and tumor necrosis factor-related apoptosis-inducing ligand . | |  |
| BNIP3L | BNIP3 and BNIP3L are pro-apoptotic members of the Bcl-2 family functioning simultoneausly. Actively involved in promoting apoptosis by inducing the expression of death receptor ligands, including Fas ligand and tumor necrosis factor-related apoptosis-inducing ligand . | |  |
| **ITGB3BP** | Pro-apoptotic | |  |
| **RASSF1** | **RASSF1** elicits apoptosis through an MST2 pathway directing proapoptotic transcription by the p73 tumor suppressor protein. Silencing of RASSF1A in tumors removes a proapoptotic signal emanating from p73 . | |  |
| TP53 | Pro-apoptotic | |  |
| - ***Genes that are present and active in lupus cells those related to anti-apoptotic function*** | | | |
| **BHLHB2**  STRA13 | Stra13 is highly expressed in unstimulated, resting B cells and is rapidly downregulated by a variety of stimuli that activate B cells, including CD40 ligand, anti-IgM antibodies, lipopolysaccharides and interleukin-4. Forced expression of Stra13 in B cells delayed the cell cycle progression into S phase and strongly suppressed Fas-mediated apoptosis . Antagonist of serum deprivation-induced apoptosis . | |  |
| **IKKA** | IKKalpha regulates the non-canonical pathway involved in lymphoid organogenesis and B-cell development; is critical for induction of pro-inflammatory and anti-apoptotic gene profiles . | |  |
| IER3  IEX1 | IEX1 overexpression considerably reduces apoptosis . | |  |
| NFKB2 | NFKB2 protects cancer cells against apoptosis . | |  |
| 1. **Other genes** **also demonstrating significant differences in dynamics but not associated with above presented groups.** Their reproducible and very distinctive behavior deserves careful further investigation. | | | |
| - ***Genes overactive in healthy control samples and stabile or not expressed in lupus patient cells*** | | | |
| HMGCR | Normally in mammalian cells this enzyme is suppressed by cholesterol derived from the internalization and degradation of low density lipoprotein (LDL) via the LDL receptor. Competitive inhibitors of the reductase induce the expression of LDL receptors in the liver, which in turn increases the catabolism of plasma LDL and lowers the plasma concentration of cholesterol, an important determinant of atherosclerosis. | |  |
| **HMGCL** | HMGCR, HMGCL – involved in biosynthesis of steroids, demonstrate negative association with LDLR. Involved in atherosclerosis, Crohn’s disease and other pathologies associated with cholesterol synthesis. | |  |
| **ZBTB25**  ZNF46, KUP | DNA binding protein. The highest levels of expression of the gene were found in testes, fetal liver, and hematopoietic cells. Its structure, resembling that of the Kruppel family, suggests that it is also a transcription factor. The precise function and DNA motif recognized by the KUP protein remain to be determined. | |  |
| **AICDA** | Activation-induced deaminase initiates somatic hypermutation - introduces point mutations into immunoglobulin genes in germinal centre B cells during an immune response. The observed here over-expression of the AICDA can be associated with EBV infection of B cells | |  |
| **PGF** | Li et al. reported experiments bearing on the therapeutic potential of PLGF and its receptor FLT1 in angiogenesis. FLT1 has anti-inflammatory effect .  Thus, PGF and FLT1 were considered potential candidates for therapeutic modulation of angiogenesis and inflammation . | |  |
| **HIG2** |  | |  |
| **P2Y5** |  | |  |
| **HRSP12** |  | |  |
| ZNF331 |  | |  |
| **GCHFR** |  | |  |
| **LGMN** | The mammalian legumain, also called asparaginyl endopeptidase (AEP), is critically involved in the processing of bacterial antigens for MHC class II presentation. Critical role for AEP in lysosomal antigen degradation . | |  |
| - ***Genes overactive in lupus samples and stabile or not expressed in normal controls*** | | | |
| ICAM1 | ICAM1 molecule is a central component of the mechanism of lymphocyte-endothelial cell adhesion. The studies of lymphoid function demonstrate a pivotal role for this molecule in both the T-cell/T-cell and T-cell/B-cell interactions, which underpin the regulation of the immune response, and in the mechanism of cell-mediated cytotoxicity. | |  |
| **GPR137B**  TM7SF1 | TM7SF1, a gene that is transcriptionally upregulated during kidney development . | |  |
| LDLR |  | |  |
| NAB1 | EGR1 binding protein | |  |
| MYO5A | myosin V function in the centrosome of human lymphocytes may be essential either for cellular proliferation or for the polarized movement of the centrosome that occurs during T-killer or T-helper cell response. | |  |
| RAB7L1 |  | |  |
| GYS1 |  | |  |
| TCIRG1 |  | |  |

**REFERENCES**

1. Glynne R, Ghandour G, Rayner J, Mack DH, Goodnow CC (2000) B-lymphocyte quiescence, tolerance and activation as viewed by global gene expression profiling on microarrays. Immunol Rev 176: 216-246.

2. Newton JS, Li J, Ning ZQ, Schoendorf DE, Norton JD, et al. (1996) B cell early response gene expression coupled to B cell receptor, CD40 and interleukin-4 receptor co-stimulation: evidence for a role of the egr-2/krox 20 transcription factor in B cell proliferation. Eur J Immunol 26: 811-816.

3. Dickinson JA, Amato SF, McManus BJ, Chiles TC (1995) Membrane immunoglobulin receptor cross-linking induces fosB mRNA expression in mature B lymphocytes: assembly of distinct FosB-containing nucleoprotein complexes during B cell stimulation. Cell Immunol 165: 92-100.

4. Huo L, Rothstein TL (1995) Receptor-specific induction of individual AP-1 components in B lymphocytes. J Immunol 154: 3300-3309.

5. Scandella E, Fink K, Junt T, Senn BM, Lattmann E, et al. (2007) Dendritic cell-independent B cell activation during acute virus infection: a role for early CCR7-driven B-T helper cell collaboration. J Immunol 178: 1468-1476.

6. Johnson BA, Geha M, Blackwell TK (2000) Similar but distinct effects of the tristetraprolin/TIS11 immediate-early proteins on cell survival. Oncogene 19: 1657-1664.

7. Frasca D, Landin AM, Riley RL, Blomberg BB (2008) Mechanisms for decreased function of B cells in aged mice and humans. J Immunol 180: 2741-2746.

8. Romzova M, Hohenadel D, Kolostova K, Pinterova D, Fojtikova M, et al. (2006) NFkappaB and its inhibitor IkappaB in relation to type 2 diabetes and its microvascular and atherosclerotic complications. Hum Immunol 67: 706-713.

9. Jeffrey KL, Brummer T, Rolph MS, Liu SM, Callejas NA, et al. (2006) Positive regulation of immune cell function and inflammatory responses by phosphatase PAC-1. Nat Immunol 7: 274-283.

10. Jonsen A, Bengtsson AA, Nived O, Truedsson L, Sturfelt G (2007) Gene-environment interactions in the aetiology of systemic lupus erythematosus. Autoimmunity 40: 613-617.

11. Yanaba K, Bouaziz JD, Matsushita T, Magro CM, St Clair EW, et al. (2008) B-lymphocyte contributions to human autoimmune disease. Immunol Rev 223: 284-299.

12. Seshasayee D, Valdez P, Yan M, Dixit VM, Tumas D, et al. (2003) Loss of TACI causes fatal lymphoproliferation and autoimmunity, establishing TACI as an inhibitory BLyS receptor. Immunity 18: 279-288.

13. Palmesino E, Moepps B, Gierschik P, Thelen M (2006) Differences in CXCR4-mediated signaling in B cells. Immunobiology 211: 377-389.

14. Watanabe T, Suzuki J, Mitsuo A, Nakano S, Tamayama Y, et al. (2008) Striking alteration of some populations of T/B cells in systemic lupus erythematosus: relationship to expression of CD62L or some chemokine receptors. Lupus 17: 26-33.

15. Henneken M, Dorner T, Burmester GR, Berek C (2005) Differential expression of chemokine receptors on peripheral blood B cells from patients with rheumatoid arthritis and systemic lupus erythematosus. Arthritis Res Ther 7: R1001-1013.

16. Klinger M, Szewczyk Z, Robak M (1983) Aldolase and adenosine deaminase activity in lymphocytes of patients with glomerulonephritis. Int Urol Nephrol 15: 273-279.

17. Aressy B, Bugler B, Valette A, Biard D, Ducommun B (2008) Moderate variations in CDC25B protein levels modulate the response to DNA damaging agents. Cell Cycle 7: 2234-2240.

18. Zornig M, Evan GI (1996) Cell cycle: on target with Myc. Curr Biol 6: 1553-1556.

19. Epeldegui M, Hung YP, McQuay A, Ambinder RF, Martinez-Maza O (2007) Infection of human B cells with Epstein-Barr virus results in the expression of somatic hypermutation-inducing molecules and in the accrual of oncogene mutations. Mol Immunol 44: 934-942.

20. Hua J, Kirou K, Lee C, Crow MK (2006) Functional assay of type I interferon in systemic lupus erythematosus plasma and association with anti-RNA binding protein autoantibodies. Arthritis Rheum 54: 1906-1916.

21. Nikiforov MA, Chandriani S, Park J, Kotenko I, Matheos D, et al. (2002) TRRAP-dependent and TRRAP-independent transcriptional activation by Myc family oncoproteins. Mol Cell Biol 22: 5054-5063.

22. Ward Y, Yap SF, Ravichandran V, Matsumura F, Ito M, et al. (2002) The GTP binding proteins Gem and Rad are negative regulators of the Rho-Rho kinase pathway. J Cell Biol 157: 291-302.

23. Taki F, Kume H, Kobayashi T, Ohta H, Aratake H, et al. (2007) Effects of Rho-kinase inactivation on eosinophilia and hyper-reactivity in murine airways by allergen challenges. Clin Exp Allergy 37: 599-607.

24. Nakamura K, Sakaue H, Nishizawa A, Matsuki Y, Gomi H, et al. (2008) PDK1 regulates cell proliferation and cell cycle progression through control of cyclin D1 and p27Kip1 expression. J Biol Chem 283: 17702-17711.

25. Houben R, Troppmair J, Hidalgo J, Rapp UR (1997) Differential gene expression in apoptotic 32Dcl3 cells: induction of metallothionein. Apoptosis 2: 40-46.

26. Miesel R, Zuber M (1993) Copper-dependent antioxidase defenses in inflammatory and autoimmune rheumatic diseases. Inflammation 17: 283-294.

27. Crowthers KC, Kline V, Giardina C, Lynes MA (2000) Augmented humoral immune function in metallothionein-null mice. Toxicol Appl Pharmacol 166: 161-172.

28. Habelhah H, Frew IJ, Laine A, Janes PW, Relaix F, et al. (2002) Stress-induced decrease in TRAF2 stability is mediated by Siah2. EMBO J 21: 5756-5765.

29. Malone MH, Wang Z, Distelhorst CW (2004) The glucocorticoid-induced gene tdag8 encodes a pro-apoptotic G protein-coupled receptor whose activation promotes glucocorticoid-induced apoptosis. J Biol Chem 279: 52850-52859.

30. Nishioka E, Funasaka Y, Kondoh H, Chakraborty AK, Mishima Y, et al. (1999) Expression of tyrosinase, TRP-1 and TRP-2 in ultraviolet-irradiated human melanomas and melanocytes: TRP-2 protects melanoma cells from ultraviolet B induced apoptosis. Melanoma Res 9: 433-443.

31. Chen J, Hui ST, Couto FM, Mungrue IN, Davis DB, et al. (2008) Thioredoxin-interacting protein deficiency induces Akt/Bcl-xL signaling and pancreatic beta-cell mass and protects against diabetes. FASEB J 22: 3581-3594.

32. Aisaki K, Aizawa S, Fujii H, Kanno J, Kanno H (2007) Glycolytic inhibition by mutation of pyruvate kinase gene increases oxidative stress and causes apoptosis of a pyruvate kinase deficient cell line. Experimental hematology 35: 1190-1200.

33. Gong W, Suzuki K, Russell M, Riabowol K (2005) Function of the ING family of PHD proteins in cancer. Int J Biochem Cell Biol 37: 1054-1065.

34. Fu Z, Tindall DJ (2008) FOXOs, cancer and regulation of apoptosis. Oncogene 27: 2312-2319.

35. Das S, Nwachukwu JC, Li D, Vulin AI, Martinez-Caballero S, et al. (2007) The nuclear receptor interacting factor-3 transcriptional coregulator mediates rapid apoptosis in breast cancer cells through direct and bystander-mediated events. Cancer Res 67: 1775-1782.

36. Matallanas D, Romano D, Yee K, Meissl K, Kucerova L, et al. (2007) RASSF1A elicits apoptosis through an MST2 pathway directing proapoptotic transcription by the p73 tumor suppressor protein. Mol Cell 27: 962-975.

37. Seimiya M, Bahar R, Wang Y, Kawamura K, Tada Y, et al. (2002) Clast5/Stra13 is a negative regulator of B lymphocyte activation. Biochem Biophys Res Commun 292: 121-127.

38. Li Y, Zhang H, Xie M, Hu M, Ge S, et al. (2002) Abundant expression of Dec1/stra13/sharp2 in colon carcinoma: its antagonizing role in serum deprivation-induced apoptosis and selective inhibition of procaspase activation. Biochem J 367: 413-422.

39. May MJ, Madge LA (2007) Caspase inhibition sensitizes inhibitor of NF-kappaB kinase beta-deficient fibroblasts to caspase-independent cell death via the generation of reactive oxygen species. J Biol Chem 282: 16105-16116.

40. Gonzalez S, Perez-Perez MM, Hernando E, Serrano M, Cordon-Cardo C (2005) p73beta-Mediated apoptosis requires p57kip2 induction and IEX-1 inhibition. Cancer Res 65: 2186-2192.

41. Viatour P, Bentires-Alj M, Chariot A, Deregowski V, de Leval L, et al. (2003) NF- kappa B2/p100 induces Bcl-2 expression. Leukemia 17: 1349-1356.

42. Li X, Tjwa M, Moons L, Fons P, Noel A, et al. (2005) Revascularization of ischemic tissues by PDGF-CC via effects on endothelial cells and their progenitors. J Clin Invest 115: 118-127.

43. Luttun A, Tjwa M, Carmeliet P (2002) Placental growth factor (PlGF) and its receptor Flt-1 (VEGFR-1): novel therapeutic targets for angiogenic disorders. Ann N Y Acad Sci 979: 80-93.

44. Burster T, Beck A, Tolosa E, Marin-Esteban V, Rotzschke O, et al. (2004) Cathepsin G, and not the asparagine-specific endoprotease, controls the processing of myelin basic protein in lysosomes from human B lymphocytes. J Immunol 172: 5495-5503.

45. Spangenberg C, Winterpacht A, Zabel BU, Lobbert RW (1998) Cloning and characterization of a novel gene (TM7SF1) encoding a putative seven-pass transmembrane protein that is upregulated during kidney development. Genomics 48: 178-185.
